# Inhibition of *Agrobacterium tumefaciens* Growth and Biofilm Formation by Tannic Acid

**DOI:** 10.3390/biomedicines10071619

**Published:** 2022-07-06

**Authors:** Afreen Jailani, Bilal Ahmed, Jin-Hyung Lee, Jintae Lee

**Affiliations:** School of Chemical Engineering, Yeungnam University, 280 Daehak-ro, Gyeongsan 38541, Korea; 22140160afreen@yu.ac.kr (A.J.); bilal22000858@yu.ac.kr (B.A.); jinhlee@ynu.ac.kr (J.-H.L.)

**Keywords:** *Agrobacterium tumefaciens*, biofilm, tannic acid, virulence, quantitative reverse transcription PCR

## Abstract

*Agrobacterium tumefaciens* underlies the pathogenesis of crown gall disease and is characterized by tumor-like gall formation on the stems and roots of a wide variety of economically important plant species. The bacterium initiates infection by colonizing and forming biofilms on plant surfaces, and thus, novel compounds are required to prevent its growth and biofilm formation. In this study, we investigated the ability of tannic acid, which is ubiquitously present in woody plants, to specifically inhibit the growth and biofilm formation of *A. tumefaciens*. Tannic acid showed antibacterial activity and significantly reduced the biofilm formation on polystyrene and on the roots of *Raphanus sativus* as determined by 3D bright-field and scanning electron microscopy (SEM) images. Furthermore, tannic acid dose-dependently reduced the virulence features of *A. tumefaciens*, which are swimming motility, exopolysaccharide production, protease production, and cell surface hydrophobicity. Transcriptional analysis of cells (Abs600 nm = 1.0) incubated with tannic acid for 24 h at 30 °C showed tannic acid most significantly downregulated the *exoR* gene, which is required for adhesion to surfaces. Tannic acid at 100 or 200 µg/mL limited the iron supply to *A. tumefaciens* and similarly reduced the biofilm formation to that performed by 0.1 mM EDTA. Notably, tannic acid did not significantly affect *R. sativus* germination even at 400 µg/mL. The findings of this study suggest that tannic acid has the potential to prevent growth and biofilm formation by *A. tumefaciens* and thus infections resulting from *A. tumefaciens* colonization.

## 1. Introduction

Biofilm formation on plant surfaces is of considerable importance, as in some cases, biofilms can promote plant growth, whereas in others, they can lead to infections [1,2]. *Agrobacterium tumefaciens* is a soil-borne, Gram-negative bacterium that thrives in the rhizosphere and readily forms biofilms and thus colonizes plant surfaces [3]. *A. tumefaciens* exerts its pathogenicity by transferring T-DNA from its tumor-inducing plasmid to host plant cells [4,5,6]. Studies have established that various strains of the genus *A. tumefaciens* are responsible for the neoplastic condition of plants referred to as crown gall [7]. The host range of *A. tumefaciens* is extensive, and it has been reported to cause crown gall disease in 643 plants belonging to 331 genera [8]. Not surprisingly, many of these host plants, including those in the Vitaceae and Rosaceae families and the genus Juglans, are economically valuable [7], and thus, *A. tumefaciens* infections result in huge financial losses due to reduced crop production [9,10].

*A. tumefaciens* is a pervasive component of soil microbiota [11] that colonizes wounded parts of plants. Amino acids, sugars, and organic acids released from wounds act as chemoattractants for the bacterium, which reaches wounds by chemotaxis and attaches firmly to cellulose fibrils [3]. Infection commences with the horizontal transmission of T-DNA present in Ti-plasmid to host cells. *A. tumefaciens* efficiently locomotes using multiple flagella positioned around a single pole [12]. The VirA/VirG system of *A. tumefaciens*, called the two-component regulatory system, activates the virulence genes required for the transfer of Ti-plasmid to the host [13]. The transition of *A. tumefaciens* from free-floating planktonic cells to surface adherent biofilms is regulated by the production of exopolysaccharides (EPS), which plays key roles in surface attachment and confer structural stability.

The agents used to control bacteria-induced plant diseases are usually based on antibiotics that are expensive and have limited bioavailabilities [14]. Agrocin 84 (a Trojan Horse antibiotic) produced by *Agrobacterium radiobacter* (a genetically modified organism) offers an alternative means of disease control by inhibiting leucyl tRNA synthetase in *A. tumefaciens* and thus its ability to synthesize proteins [15,16]. This antibiotic is specifically taken up by the opine uptake system of *A. tumefaciens*, but it may also target other beneficial organisms; additionally, the genetically modified organisms like *A. radiobacter* are not permitted for usage in certain countries [17]. Thus, novel approaches are needed to prevent *A. tumefaciens* biofilm formation to successfully control crown gall disease.

**Figure 1:**
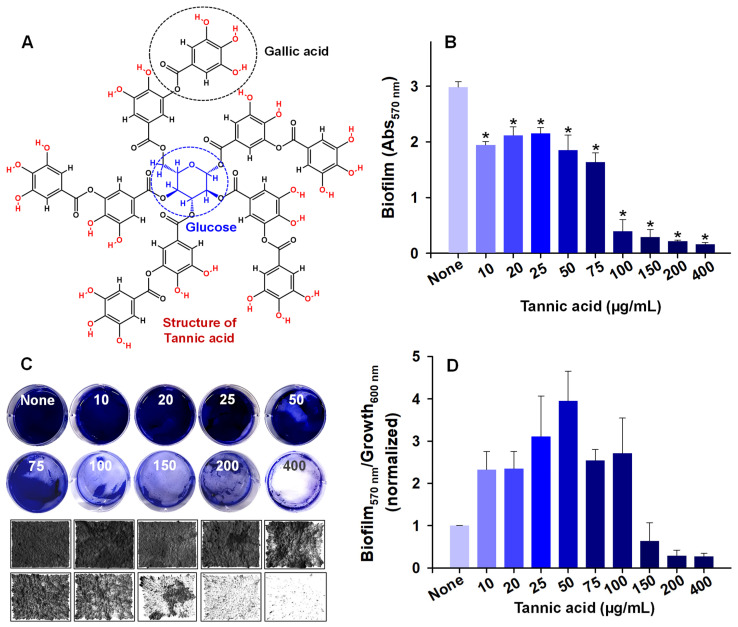
Tannic acid is composed of a central glucose with ten surrounding galloyl units (**A**). Biofilm formation of *A. tumefaciens* on polystyrene (**B**). Gross images and biofilm morphology on 6-well polystyrene plates (**C**). The normalized ratio of biofilm (Abs570 nm) to cell growth (Abs620 nm) shows tannic acid reduces biofilm formation at ≥150 µg/mL (**D**). Cells were incubated in LB containing tannic acid at the concentrations shown for 48 h at 30 °C under static conditions. Two independent bacterial colonies were used in triplicates (*n* = 6) and experiments were repeated twice. Asterisks (*) denote significant differences (*p* ≤ 0.05 by the two-tailed *t*-test) between treated groups and nontreated controls.

Polyphenols protect plants from pathogen attack and bacterial toxins [18], and tannic acid, a member of the tannin family, is one such polyphenol. Chemically, tannic acid molecules contain one glucose molecule surrounded by ten galloyl (3,4,5-trihydroxyphenyl) units (Figure 1A) [19]. Due to its presence in nearly all aerial plant tissues, it is likely that *A. tumefaciens* encounters tannic acid. In addition, tannic acid has been used traditionally to treat diarrhea and skin burns [20] and has been approved by the USA Food and Drug Administration (USFDA) as a food additive [21], and it exhibits antimicrobial activity against Gram-positive and Gram-negative bacteria [19,22]. Mechanistically, tannic acid penetrates the cell membrane and directly affects cell metabolism. More specifically, it restricts the uptakes of amino acids and sugars and thus retards bacterial growth [19]. Tannic acid has been reported to exhibit antibacterial and antibiofilm activities against *Pseudomonas aeruginosa* (at 100 µg/mL) and antibiofilm effects against *Staphylococcus aureus* (at 20 µg/mL) [23,24], and because it is a multidentate ligand, tannic acid can interact with protein sites hydrophobically or by hydrogen bonding [25,26]. In addition, tannic acid has been used as a polymer crosslinking agent, and the hydrogel produced was antibacterial to Gram-negative and -positive bacteria [27]. Furthermore, it has been suggested that the antimicrobial activity of tannic acid may be due to inhibition of the NorA efflux pump [28].

Nonetheless, the effects of tannic acid on *A. tumefaciens* growth and biofilms have not been explored, which prompted us to assess the ability of tannic acid to inhibit *A. tumefaciens* planktonic growth and biofilm formation, the major virulent traits of *A. tumefaciens* (swimming motility, EPS production, protease activity, and cell surface hydrophobicity), and its surface colonization of roots. In addition, we examined the effects of tannic acid on the expressions of 11 biofilm and virulence-related genes in *A. tumefaciens*.

## 2. Materials and Methods

### 2.1. Chemicals and Bacterial Culture

Tannic acid (Product Code 403040) was purchased from Sigma-Aldrich (St. Louis, MO, USA). *A. tumefaciens* strain GV2260 was used for all experiments. A single colony of *A. tumefaciens* was inoculated in LB medium and incubated for 24 h at 250 rpm and 30 °C. Shake cultures were prepared under similar growth conditions in flasks. Experiments were performed using at least two independent cultures (prepared from two independent colonies) in triplicate.

### 2.2. Evaluation of Biofilm Inhibition

The antibiofilm susceptibility of *A. tumefaciens* against tannic acid was analyzed using a crystal violet assay as previously described [29]. Briefly, after culturing for 24 h, *A. tumefaciens* was diluted with LB at 1:50. Tannic acid was then added in the concentration range 10–400 µg/mL, and 300 µL of these mixtures was pipetted into a 96-well plate and incubated at 30 °C for 48 h. The plate was then stained with 0.1% crystal violet for 20 min, washed with sterile water, and air-dried. Crystal violet retained by biofilms was dissolved with 300 µL of 95% ethanol, and biofilm formation was quantified by measuring absorbance (λmax) at 570 nm [29].

### 2.3. Growth Assessments of Tannic Acid Treated A. tumefaciens

Time-dependent growth of *A. tumefaciens* in the presence of tannic acid was used to investigate the ability of tannic acid to inhibit planktonic cell growth following our earlier described method [30]. After culturing for 24 h, *A. tumefaciens* was diluted with LB at 1:100, and tannic acid was added to final concentrations of 10, 20, 25, 50, 75, 100, 150, 200, or 400 µg/mL. A 300 µL aliquot of each of these cultures was then added to a 96-well plate and incubated at 30 °C for 24 h under static conditions. OD values were recorded at 620 nm every 2 h during this 24 h period, and growth curves were plotted. To determine colony-forming units (CFUs), a 24 h culture of *A. tumefaciens* was diluted with LB (1:100), and tannic acid was added (at 10–400 µg/mL) and cultured at 250 rpm for 24 h at 30 °C. Cultures were then diluted and 100 µL of each dilution was spread onto LB agar plates, which were incubated for two days at 30 °C. Colonies were then counted.

### 2.4. Growth and Biofilm Inhibition under Iron-Limiting Conditions

To check the role of ten galloyl groups of tannic acid in creating iron-limiting conditions and thus inhibition of growth and biofilms, 100 and 200 µg/mL of tannic acid were used with various concentrations of an iron source (FeSO_4_·7H_2_O). An earlier method [31] was used with modifications. The well-known iron chelator EDTA was first tested to know its threshold concentration that reduces the growth and biofilm of *A. tumefaciens* in LB. Various concentrations of EDTA (0.01, 0.02, 0.05, 0.1, 0.2, 0.5, and 1 mM) were checked on growth and biofilm formation of *A. tumefaciens* in 96-well plates under the same incubation conditions as described earlier (Section 2.2 and Section 2.3). Biofilm inhibitory concentration of EDTA was then tested with varying doses (0.01–1 mM) of Fe^2+^ (FeSO_4_·7H_2_O) in 96-well plates. In a similar manner, two concentrations of tannic acid (100 and 200 µg/mL) were tested in two different experiments with varying concentrations of FeSO_4_·7H_2_O (0.01–1 mM), and the growth and biofilm formation were checked after 48 h at 30 °C.

### 2.5. Swimming Motility Assay

Swimming motilities of *A. tumefaciens* grown in the presence of various doses of tannic acid were investigated using motility agar plates. The agar constituted 1% peptone, 0.5% NaCl, and 0.25% agarose. Before pouring into Petri plates, the agar was thoroughly mixed with different concentrations of tannic acid. Aliquots (1 µL) of 24 h cultures were placed at the centers of agar plates and incubated at 30 °C for 72 h. Swimming diameters were recorded at every 48 h and 72 h during incubation [30].

### 2.6. Estimation of EPS Released under Tannic Acid Stress

Amounts of EPS released by *A. tumefaciens* cells were estimated using a standard phenol-sulfuric acid assay, as previously described [32]. *A. tumefaciens* cells (OD600 nm = 0.5) were grown in the presence or absence of tannic acid at different concentrations in 1.5 mL microfuge tubes at 250 rpm for 24 h at 30 °C. After incubation, tubes were centrifuged for 10 min at 13,000 rpm, and supernatants were collected and diluted with chilled ethanol at 1:3. The tubes were then allowed to stand at 4°C overnight, and precipitates obtained were dissolved in 1 mL of distilled water. Phenol (5%) and concentrated sulfuric acid in the ratio of 1:5 was added to the dissolved EPS and incubated for 30 min at room temperature. Absorbances were recorded at 490 nm.

### 2.7. Cell Surface Hydrophobicity Assay

*A. tumefaciens* cells (OD600 nm = 0.5) were grown for 24 h in the presence of various concentrations of tannic acid in 1.5 mL microfuge tubes at 30 °C and 250 rpm. Tubes were then centrifuged for 10 min at 13,000 rpm. The pellets obtained were washed three times and resuspended in 1 mL of sterile PBS, and OD was measured at 600 nm (denoted A_0_). Xylene (250 µL) was added to suspensions, vortexed vigorously, and left undisturbed for 30 min. Aqueous phases were then carefully removed, and absorbances (Ai) were measured at 600 nm. Percent hydrophobicities were then calculated using the following formula [33]:

Percent cell surface hydrophobicity (% CSH) = (A_0_ − Ai)/Ai × 100

### 2.8. Effect of Tannic Acid on Protease Production

Bacterial exoprotease production was quantified as previously described [34] in the presence or absence of tannic acid. *A. tumefaciens* was grown with 25, 50, 75, 100, 150, or 200 µg/mL of tannic acid at 250 rpm for 24 h at 30 °C. Microfuge tubes were centrifuged for 10 min at 13,000 rpm, and supernatant (100 µL) was collected in fresh microfuge tubes, treated with 100 µL of azocasein for 30 min at 37°C, and then with 600 µL of trichloroacetic acid (10%) to halt proteolysis. The tubes were then cooled at −20 °C for 30 min, and 700 µL of 1 M NaOH was added. Absorbances were recorded at 440 nm.

### 2.9. Visualization of A. tumefaciens Biofilms on Nitrocellulose Membranes and R. sativus Roots by Scanning Electron Microscopy (SEM)

The biofilms of *A. tumefaciens* were formed on 3 × 3 mm nitrocellulose membranes in a 96-well plate. Bacterial culture diluted with LB (1:50) and treated with 200 µg/mL of tannic acid was pipetted into wells and allowed to grow for 48 h at 30 °C. To investigate biofilm development on roots and the effects of tannic acid, surface-sterilized seeds of *R. sativus* were placed on soft agar plates (0.7% agar + 0.86 g/L Murashige and Skoog plant growth medium) and grown for four days. At least five seedlings from control and treated groups were placed in 6-well plates containing 200 µg/mL of tannic acid in inoculum of *A. tumefaciens* (1:50 dilution) and incubated at 30 °C for 48 h. Roots were then gently rinsed once with PBS. Before SEM imaging, samples (nitrocellulose membranes and roots) were processed as follows: (i) they were fixed in a mixture of 2.5% glutaraldehyde and 2% paraformaldehyde for 30 min at room temperature and overnight at 4 °C, and (ii) samples were dehydrated using an ethanol series (30%, 50%, 70%, 90%, and 100% for 10 min each), (iii) preserved in isoamyl acetate, (iv) critical point dried and sputter coated with gold or platinum, and (v) visualized at different magnifications using a S-4200 Hitachi Field Emission (FE) SEM microscope (Hitachi Systems Ltd., Tokyo, Japan) at 15 kV.

### 2.10. Potato Disc Antitumor Assay

An earlier described method for potato disc tumor formation was followed [35] with slight modifications. Potato discs were infected by *A. tumefaciens* and checked after 3 weeks (incubation at 25 °C) for the development of tumors. Potatoes were surface cleaned with tap water thoroughly to remove all the dirt, followed by surface cleaning with ethanol (70%). Potatoes were cut to obtain flat cubes. Thick slices were made using the sterile knife and immersed in NaOCl (6%) for 15 min. Potato discs (1 cm in diameter) were cut from the middle part of cubes using a sterile cork borer. Discs were rinsed thrice in sterile distilled water. For infection, *A. tumefaciens* culture (OD600 nm = 1.0) was exposed to tannic acid (200 µg/mL) in PBS and vortexed. A 50 µL of the cell suspension was added to the surface of each potato disc and placed in 6-well tissue culture plates. Plates were sealed and incubated at 25 °C for three weeks in the dark. The numbers of *A. tumefaciens* induced tumors that appeared on potato surfaces were counted, and percent tumor induction was calculated.

$$Tumor\;induction\;\left(\%\right)=Number\;of\;tumors\;in\;\frac{treated\;samples}{Untreated\;control}\times 100$$

### 2.11. Effects of Tannic Acid on Seed Germination

*R. sativus* seeds were rinsed with distilled water three times and once with 95% ethanol and surface disinfected by emersion in 3% sodium hypochlorite for 10 min. Seeds (*n* = 10) were then placed on agar plates containing different concentrations of tannic acid (25–400 µg/mL), 0.7% agar, and 0.86 g/L Murashige and Skoog plant growth medium [30]. Plates were then incubated for four days at 25 °C, and percent seed germinations were calculated with respect to nontreated controls.

### 2.12. RNA Extraction and Gene Expression Analysis by qRT-PCR

To determine whether tannic acid treatment affected the expression levels of genes related to biofilm, stress, motility, and virulence, *A. tumefaciens* was grown in the presence or absence of tannic acid. *A. tumefaciens* culture in 25 mL LB having an OD of 1.0 at 600 nm was added with 200 µg/mL of tannic acid and kept at 250 rpm for 24 h at 30 °C, then 700 µL of RNase inhibitor (RNAlater, Ambion, TX, USA) was added with gentle mixing [23]. Cultures were maintained under chilled conditions in an ice bath to prevent RNA degradation and harvested by centrifugation at 13,000 rpm for 10 min at 4 °C. RNAs from cell pellets were extracted using the Qiagen RNeasy mini kit (Valencia, CA, USA), and RNA purities were checked using a nanodrop spectrophotometer (Cytiva NanoVue Plus Spectrophotometer, Fisher Scientific, Loughborough, UK). Quantitative reverse transcription real time polymerase chain reaction (qRT-PCR) was carried out to assess the expressions of genes responsible for biofilm formation (*celA*, *cheA*, *exoR*, *phoB*), virulence (*chvE*, *chvG*), motility (*flgE*, *fliR*, *motA*), and stress response (*clpB*, *dnaK*). Gene functions and primer sequences are given in Supplementary Table S1. The 16S rRNA gene was used as the housekeeping gene, and the reaction was carried out using SYBR green master mix and the ABI StepOne Real-Time PCR System, Applied Biosystems, Foster City, CA, USA [23].

### 2.13. Statistical Analysis

Two independent bacterial colonies were used for experiments in triplicates, and results are presented as means ± standard deviations. Values of *p* ≤ 0.05 were considered significant as per Student’s *t*-test. All statistical analyses were performed, and graphs were plotted on SigmaPlot Windows Version 14.0 (Build 14.0.0.124), Systat Software, Inc., Chicago, IL, USA. Asterisks in figures indicate significant changes versus nontreated controls.

## 3. Results

### 3.1. Tannic Acid Inhibited Biofilm Formation by A. tumefaciens

**Figure 2:**
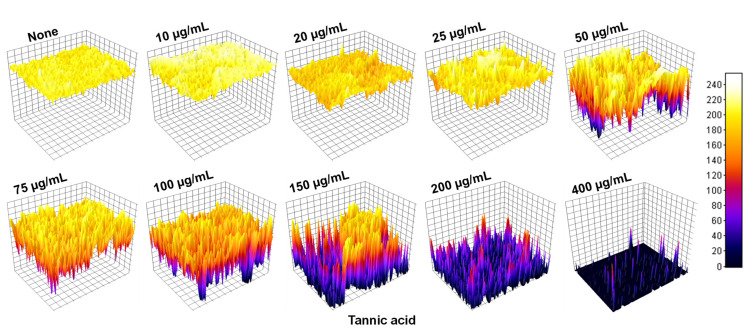
The 3D-thermal LUT plots demonstrating concentration-dependent biofilm formation on flat polystyrene surfaces by nontreated and tannin-treated *A. tumefaciens*. Biofilms were formed on polystyrene surface in 6-well plates in LB with or without tannic acid for 48 h at 30 °C under static conditions. Crystal violet (0.1%) stained biofilms (for 20 min) were imaged using iRisTM Digital Cell imaging, Logos Biosystems, Annandale, VA, USA. The color-coded pictures correspond to the scale (right side of the image) where zero shows negligible biofilm and 240 represents maximum formation of biofilm. Two independent bacterial colonies were used in triplicates (*n* = 6), and experiments were repeated twice.

Tannic acid (Figure 1A) in the range 10–400 µg/mL dose-dependently and significantly inhibited *A. tumefaciens* biofilm formation (Figure 1B). Tannic acid at 100 µg/mL inhibited biofilm formation by 86.6%, and this inhibition increased further on increasing tannic acid concentration (e.g., to 92.7% at 200 µg/mL). Bright-field microscopy (Figure 1C) showed slight biofilm formation at 100 µg/mL and little evidence of biofilm formation at higher concentrations. The normalized data of biofilm (570 nm) to growth (620 nm) first increased up to 100 µg/mL, which then decreased sharply at 150 µg/mL and further reduced at 200 µg/mL (Figure 1D). This suggests that apart from bacteriostatic/bactericidal activity, tannic acid also interferes with the biofilm formation of *A. tumefaciens* and reduces it. Micrographs of biofilms were recreated as three-dimensional LUT mesh plots (Figure 2). Colors and color intensities of biofilms produced with or without tannic acid showed slight biofilm formation at 50–100 µg/mL but almost no biofilm formation at 200 µg/mL. Biofilm quantification and their visualization on polystyrene confirmed the inhibitory potential of tannic acid.

### 3.2. Tannic Acid Compromised the Growth and Viability of A. tumefaciens

**Figure 3:**
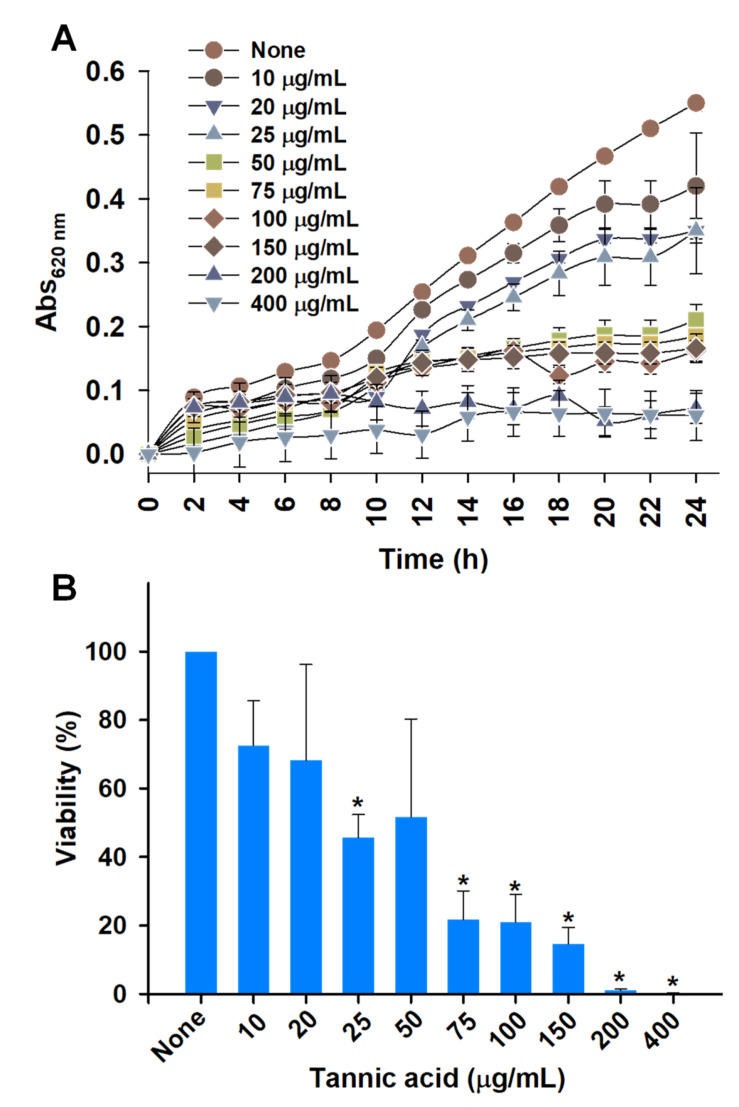
Growth inhibition of *A. tumefaciens* by tannic acid cultured in LB for 24 h at 30 °C. Growth curves (**A**) and percent cell viability (**B**) after exposure to tannic acid. Two independent bacterial colonies were used in triplicates (*n* = 6) and experiments were repeated twice. ‘*’ denotes significant difference between nontreated and treated *A. tumefaciens*. *p* ≤ 0.05 as determined by the two-tailed *t*-test.

Tannic acid has antibacterial activity against several clinical pathogens like *S. aureus*, *P. aeruginosa*, *Escherichia coli*, *Streptococcus pyogenes*, *Enterococcus faecalis*, and *Listeria innocua* [19]. We found tannic acid dose-dependently delayed the growth of *A. tumefaciens* (Figure 3A). At concentrations from 50 to 150 µg/mL and incubation for 8 h, tannic acid had similar inhibitory effects on *A. tumefaciens* growth. In addition, tannic acid dose-dependently reduced cell viabilities (Figure 3B). At 75 µg/mL, tannic acid remarkably inhibited cell viability (> 70%), and at 200 µg/mL, few cells survived.

### 3.3. External Iron Supply Restored the Growth and Biofilm of A. tumefaciens

**Figure 4:**
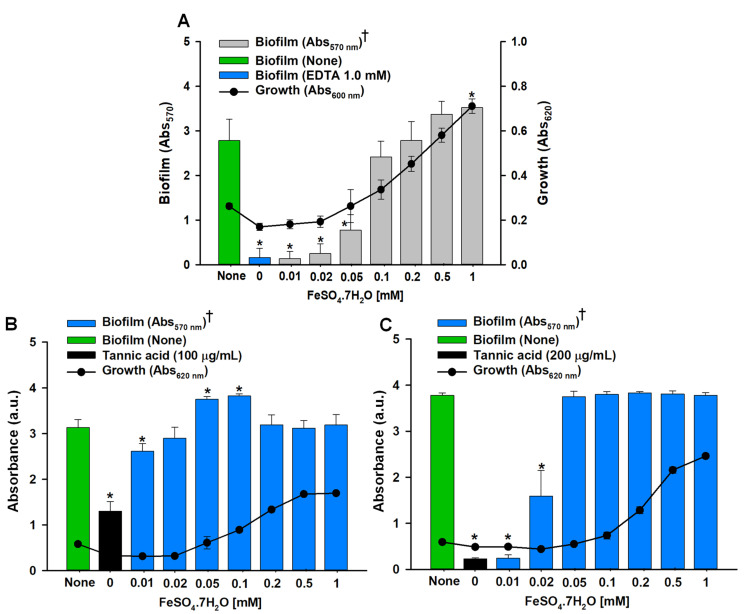
Effect of iron limitation on growth and biofilm formation of *A. tumefaciens*. Line and scatter plots represent cell growth absorbance at 620 nm and bars show the amount of biofilm produced (570 nm) in 96-well microtiter plates after 48 h incubation at 30 °C in LB. Effect of increasing concentrations of iron source (Fe_2_SO_4_·7H_2_O) with constant EDTA (1 mM) (panel (**A**)), constant 100 µg/mL tannic acid (panel (**B**)), and 200 µg/mL tannic acid (panel (**C**)). Bar for ‘none’ shows biofilm formation without EDTA or tannic acid or Fe_2_SO_4_·7H_2_O. Biofilm (570 nm) bars marked with ‘†’ show biofilm in the presence of an iron source. Two independent bacterial colonies were used in triplicates (*n* = 6), and each experiment was repeated at least twice. Asterisks denote significant differences between ‘none’ and treated *A. tumefaciens* at *p* ≤ 0.05 by the two-tailed *t*-test.

The EDTA (1 mM) significantly reduced the biofilm formation and growth of *A. tumefaciens* growing in LB with no supply of external iron (Fe_2_SO_4_·7H_2_O) (Figure 4A). When Fe_2_SO_4_·7H_2_O (0.01–1 mM) was supplied with 1 mM EDTA in LB, the growth and biofilm formation started to resume at 0.02 mM Fe_2_SO_4_·7H_2_O and increased in an iron concentration-dependent manner (Figure 4A). This suggests that iron was one of the limiting factors for the growth and biofilm of *A. tumefaciens*. Similarly, when tannic acid (100 and 200 µg/mL) was used instead of EDTA (1 mM), it substantially reduced the biofilm in the absence of external iron supply (Figure 4B,C). The Fe_2_SO_4_·7H_2_O restored the biofilm formation at 0.01 mM in the presence of 100 µg/mL tannic acid (Figure 4B), while 0.02 mM Fe_2_SO_4_·7H_2_O was required to mitigate the effect of 200 µg/mL tannic acid (Figure 4C). At ≥0.05 mM Fe_2_SO_4_·7H_2_O, the biofilm of *A. tumefaciens* was restored completely and was found equivalent to nontreated group; however, the growth increased further than the nontreated control until 1 mM Fe_2_SO_4_·7H_2_O.

### 3.4. Effects of Tannic Acid on Virulence Properties: EPS, Protease Activity, and Cell Surface Hydrophobicity (CSH)

**Figure 5:**
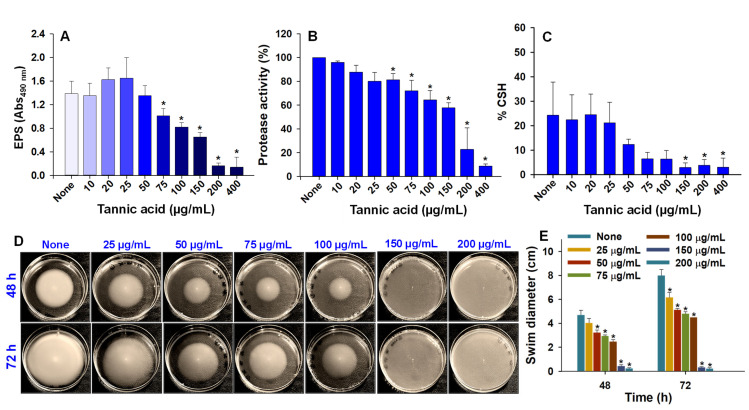
Tannic acid concentration-dependent decrease in the virulence traits of *A. tumefaciens*. Exopolysaccharides (EPS) production (**A**), protease activity (**B**), and percent cell surface hydrophobicity (%CSH) (**C**) after incubation at 250 rpm for 24 h at 30 °C in LB. Concentration-dependent effect of tannic acid on the swimming motility of *A. tumefaciens* (**D**) after incubation for 48–72 h tannic acid containing peptone agar (1% peptone, 0.5% NaCl, 0.25% agarose). Swim diameters were recorded after incubation for 48 or 72 h (**E**). For EPS production, protease activity, and %CSH, two independent bacterial colonies were used in triplicates (*n* = 6) and experiments were repeated twice. For swimming motility, three independent colonies were used (*n* = 3), and experiment was repeated twice. Asterisks denote significant differences between nontreated and treated *A. tumefaciens*. *p* ≤ 0.05 by the two-tailed *t*-test.

EPS benefits embedded bacterial cells by providing a protective shield that prevents antimicrobial ingress and enhances the structural stabilities and integrities of biofilms [36]. We examined the effect of tannic acid on EPS production by *A. tumefaciens* using a phenol-sulfuric acid assay (Figure 5A). Marginal EPS increases of 17% and 19% were observed at tannic acid concentrations of 20 and 25 µg/mL, presumably in response to tannic acid-induced stress [37]. However, at concentrations above 50 µg/mL, tannic acid significantly and dose-dependently reduced EPS production. For example, at 100 and 200 µg/mL, tannic acid reduced EPS production by 41% and 90%, respectively.

The extracellular release of proteases is another important virulence factor, as this release plays an important role in the spread of infection and can cause irreparable host damage [38]. We found that at 50 to 400 µg/mL tannic acid dose-dependently reduced protease activity (Figure 5B), and that at 200 and 400 µg/mL, it caused reductions of 77.3% and 91.3%, respectively.

The CSH is also an important virulence factor, as it influences the abilities of cells to adhere to biotic and abiotic surfaces. Tannic acid decreased %CSH significantly at concentrations ≥150 µg/mL (Figure 5C), after which its effect plateaued.

### 3.5. Swimming Motility of A. tumefaciens Exposed to Tannic Acid

Swimming motility plays a central role in bacterial biofilm formation and in the pathogeneses of resulting diseases [12]. *A. tumefaciens* GV2260 demonstrated swimming motility on agar plates in the absence of tannic acid; maximum swim diameters of nontreated controls were 4.7 and 8 cm after incubation for 48 h and 72 h, respectively. However, tannic acid treatments concentration-dependently reduced swimming motility (Figure 5D,E), and its effect was greatest at 100 µg/mL, at which swim diameters were 2.4 and 4.5 cm after incubation for 48 h and 72 h, respectively. At concentrations of 150 and 200 µg/mL incubation with tannic acid for 48 h completely inhibited swimming motility.

### 3.6. Tannic Acid Inhibited Biofilm Formation on Nitrocellulose and on the Roots of R. sativus

**Figure 6:**
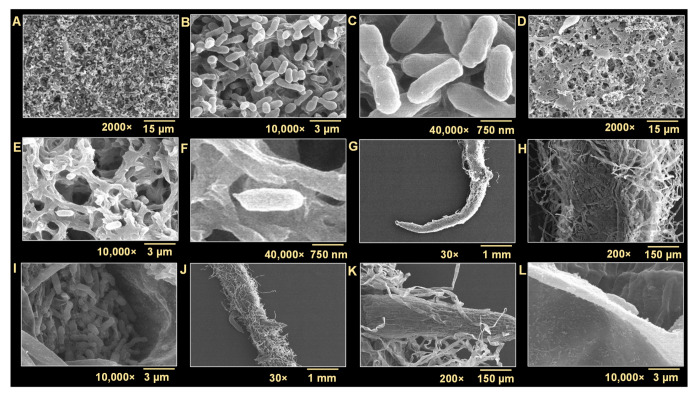
*A. tumefaciens* biofilm formation (48 h at 30 °C in LB) by nontreated cells (**A**–**C**) and tannic acid (200 µg/mL) treated cells (**D**–**F**) on nitrocellulose membranes. Biofilm formation by *A. tumefaciens* on the roots of *R. sativus* in the absence (**G**–**I**) and presence of tannic acid at 200 µg/mL (**J**–**L**) as visualized by scanning electron microscopy (SEM) at different magnifications. Two independent bacterial colonies were used in triplicates (*n* = 6), and at least five micrographs captured from five different random locations for each sample were analyzed.

Biofilms on nitrocellulose membranes and plant roots in the presence and absence of tannic acid were visualized by SEM. Comparisons of micrographs of biofilms formed in the absence (Figure 6A–C) and presence of tannic acid (Figure 6D–F) showed that biofilm formation on nitrocellulose membranes was severely inhibited by tannic acid at 200 µg/mL. In the absence of tannic acid, biofilms were dense, well-structured, and uniform on nitrocellulose, but only a few scattered cells of *A. tumefaciens* were found in the presence of tannic acid. However, despite the obvious inhibition of biofilm formation by tannic acid, the only morphological difference noticed was a slight reduction in cell length.

Biofilm formation on the roots of *R. sativus* was examined after incubation for 48 h with *A. tumefaciens* in the presence or absence of tannic acid. SEM images provided insight into the extent of colonization of root surfaces (Figure 6G–L). Roots incubated in the absence of tannic acid showed extensive colonization by the pathogen on root hairs and surrounding areas (Figure 6G–I). On the other hand, barely any colonization was observed on the root surfaces of seedlings incubated in the presence of tannic acid at 200 µg/mL (Figure 6J–L).

### 3.7. Influence of Tannic Acid on Tumor Induction by A. tumefaciens and Seed Germination

**Figure 7:**
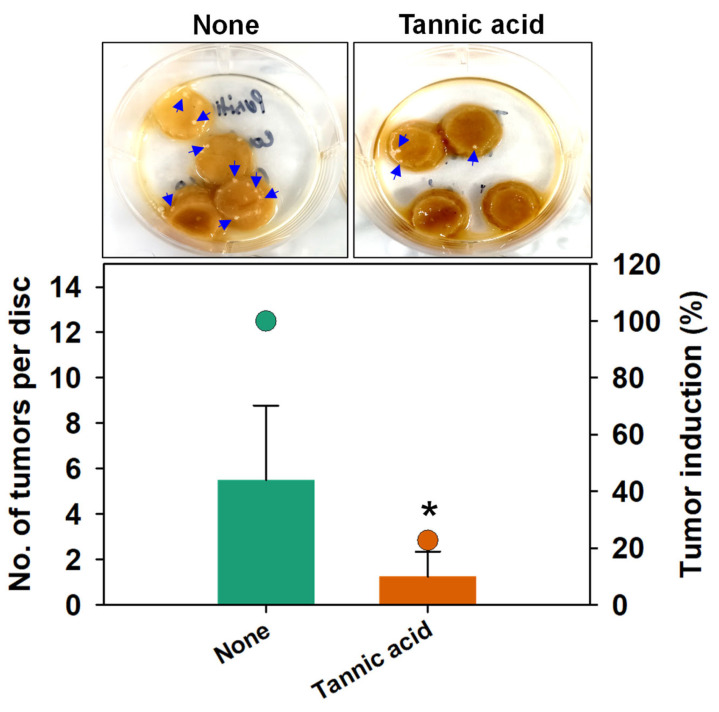
Induction of tumors by *A. tumefaciens* on potato discs and its inhibition by tannic acid (200 µg/mL). Bars show numbers of tumors per disc while symbols represent tumor induction (%). Representative pictures of potato discs with tumors marked with blue arrows are shown on the top. Two independent bacterial colonies were used in triplicates (*n* = 6), and experiment was repeated twice. ‘*’ shows a significant difference at *p* ≤ 0.05 by the two-tailed *t*-test.

**Figure 8:**
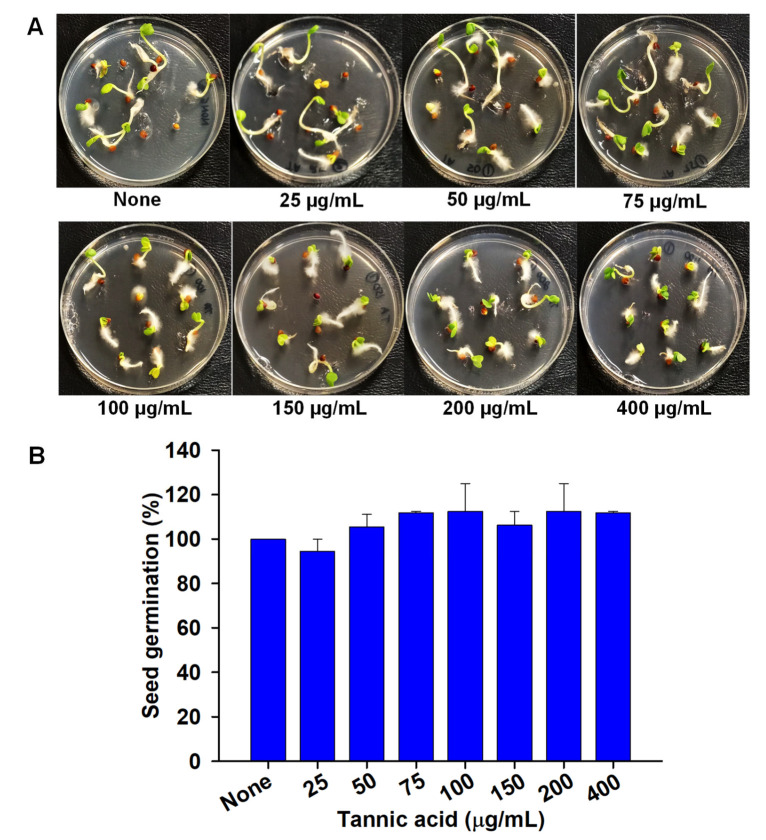
Toxicity assessment of tannic acid on *R. sativus* seed germination over 5 days on tannic acid infused into 0.7% agar and 0.86 g/L MS medium (**A**). Bar plot of percentage seed germination as a function of increasing tannic acid concentration (**B**). Seeds (*n* = 10) were used for each concentration, and experiment was repeated twice.

Tannic acid (200 µg/mL) exposed cells of *A. tumefaciens* (OD600 nm = 1.0) lost their ability to induce tumors on potato slices. The average reduction in tumor formation was 78% by tannic acid over nontreated control (Figure 7). Tannic acid was examined to determine any impact on *R. sativus* seed germination (Figure 8). *R. sativus* seeds germinated on soft agar plates (Figure 8A) containing MS plant growth solution and tannic acid (25–400 µg/mL) were checked after four days of incubation. Notably, tannic acid did not inhibit germination at any of the concentrations examined (Figure 8B).

### 3.8. Effect of Tannic Acid on A. tumefaciens Gene Expressions

**Figure 9:**
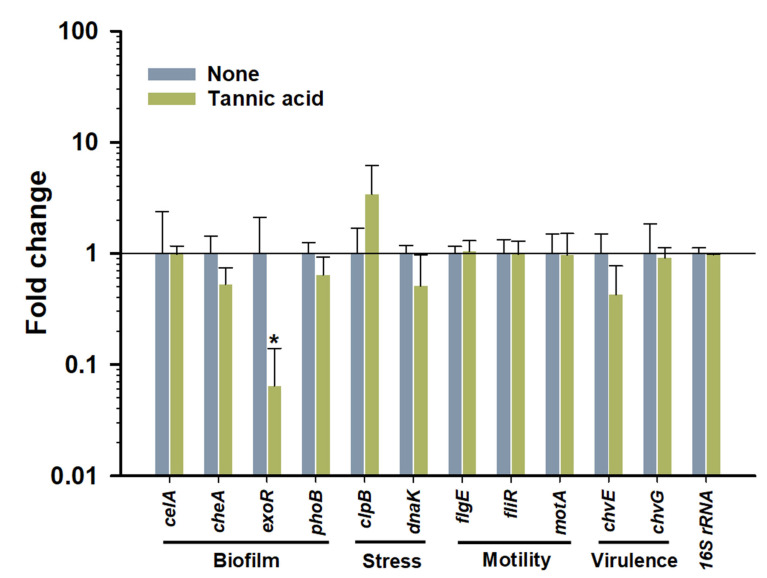
qRT-PCR analysis of the expressions of genes after treating *A. tumefaciens* with tannic acid at 200 µg/mL. The asterisk denotes a significant difference between nontreated and treated *A. tumefaciens*. Two independent bacterial colonies were used in duplicates (*n* = 4), and experiment was repeated twice. *p* ≤ 0.05 by the two-tailed *t*-test.

Certain bacterial genes influence characteristics such as biofilm formation (*celA*, *cheA*, *exoR*, *phoB*), stress response (*clpB*, *dnaK*), motility (*flgE*, *fliR*, *motA*), and virulence (*chvE*, *chvG*), and thus, we examined the effects of tannic acid at 200 µg/mL on the expressions of these genes (Figure 9). qRT-PCR showed that *exoR*, which is required for biofilm formation [39], was downregulated by >15-fold by tannic acid. Downregulation of this gene has been proposed to be a cause of compromised surface adherence to plant and abiotic surfaces [40]. With the exceptions of *dnaK* and *chvE*, which were slightly downregulated, tannic acid had no effect on the expression of other genes tested.

## 4. Discussion

*A. tumefaciens* resides in the top layer of soil and can adhere to biotic and abiotic surfaces [3,30]. The transition from sessile to surface-attached *A. tumefaciens* is determined by adhesins and flagellar motility [40]. Tannic acid is ubiquitously present in woody and herbaceous plants and possesses antimutagenic and antitumor properties and antibacterial and antiviral activities [19,41], which has generated interest in the extraction of polyphenols with unique antimicrobial properties from natural sources. Many pathogens develop resistance against conventional treatments when their planktonic forms are targeted [16], and thus, biofilm targeting is viewed as an attractive potential treatment option [42]. We investigated whether tannic acid at concentrations from 10 to 400 µg/mL could effectively inhibit *A. tumefaciens* biofilm formation and explored the effects of tannic acid on its biofilm-forming capacity, root colonizing ability, and virulence attributes. We found that tannic acid markedly reduced *A. tumefaciens* biofilm formation at 100 µg/mL (Figure 1B,C) and reduced the viability of *A. tumefaciens* cells (Figure 3). In addition, tannic acid dramatically inhibited protease secretion and EPS production by *A. tumefaciens* and its surface hydrophobicity (Figure 5A–C), cell motility (Figure 5D,E), and abilities to adhere to polystyrene or nitrocellulose (Figure 2 and Figure 6) and root surfaces (Figure 6G–L). This was possibly caused by hydrophobic and H-bonding interactions between tannic acid and bacterial proteins [19]; as has been reported, tannic acid acts as a multidentate ligand due to interactions between its aromatic rings and phenolic groups and protein surfaces [43]. Furthermore, inhibition of the NorA efflux pump by tannic acid has been proposed to be responsible for its antimicrobial effects on *Staphylococcus aureus* and *Enterococcus faecalis* [28]. NorA confers resistance against ciprofloxacin in *S. aureus* [44], and the AcrAB efflux pump has a similar function in *A. tumefaciens* and provides resistance against ciprofloxacin and other drugs [45]. Thus, it is possible that tannic acid targets AcrAB or related efflux pumps in *A. tumefaciens*.

Tannic acid significantly inhibited biofilm formation by *A. tumefaciens* (Figure 1), and previously was found to inhibit *S. aureus* biofilm formation in the presence of transglycosylase IsaA [46]. Tannic acid also effectively inhibited biofouling by *P. aeruginosa* [47]. These antibiofilm effects may be due to the high antioxidative capacity of tannic acid, as previous studies have shown that antioxidants inhibit biofilm formation by reducing the expressions of genes responsible for oxidative stress [48]. Furthermore, the EPS of *A. tumefaciens* accounts for up to 20% of its dry weight and importantly protects the bacterium from environmental stresses [49], and in the current study, tannic acid significantly and dose-dependently decreased the EPS secretion by *A. tumefaciens* from a concentration of 50 µg/mL (Figure 5A).

*A. tumefaciens* can sense chemical signals released from plant wounds and uses its swimming motility to reach these sites [12]. Swarming and twitching motilities have not been reported for this genus, and thus, swimming motility is believed to be the only means of locomotion as reported [12] and ourselves [30]. In a previous study, tannic acid inhibited the swimming motility of *C. violaceum* (a quorum-sensing-positive bacteria) at concentrations of ≥30 µg/mL [50], and in the present study, tannic acid at 25 µg/mL reduced the swimming motility of *A. tumefaciens* (Figure 5D,E). Tannic acid also impaired the other virulence traits of *A. tumefaciens*, namely protease activity and cell surface hydrophobicity (Figure 5B,C), by dose-dependently decreasing them. Likewise, tannic acid has been reported to inhibit the productions of quorum-sensing (QS) molecules and to attenuate biofilm formation by *E. coli* [51] and *Aeromonas hydrophila* [52].

The 3D images of *A. tumefaciens* biofilms on polystyrene (Figure 2) showed tannic acid dose-dependently reduced biofilm thicknesses, and this was in line with the near absence of biofilm formation on nitrocellulose membranes at a tannic acid concentration of 200 µg/mL as determined by SEM (Figure 6D–F). Plausibly, this may have been due to the inhibitory effects of tannic acid on adherence to associated genes and EPS production. Furthermore, it has also been proposed that metal ion chelation by tannic acid might cause cells to burst and thus prevent biofilm formation [53]. The ten galloyl groups of tannic acid chelate iron [54], and this process has been shown to be correlated with the antioxidant activity of tannic acid and thus to the prevention of biofilm formation [48,55]. This iron-chelating strategy has also been proposed as a means of inhibiting the activities of diverse pathogens [56]. Heindl et al. [31] suggested the possible role of manganese and iron in the surface attachment and biofilm formation of *A. tumefaciens*. They used ethylenediamine-di-o-hydroxyphenyl acetic acid (EDDHA) or 2,2′-dipyridyl (DIP) at 20–1000 µM for iron chelation, and the relative biofilm formation was inhibited dose-dependently; however, the addition of 11–1100 µM FeSO_4_ resumed the biofilm formation under the constant 200 µM concentration of either EDDHA or DIP [31]. Iron is crucially required for *A. tumefaciens* infection [57]; therefore, we assessed its role in the biofilm formation of *A. tumefaciens*. In our study, tannic acid at 100 and 200 µg/mL acted as the iron chelator similar to the EDTA (1 mM) (Figure 4). Tannic acid limited the iron supply required for the growth and biofilm formation of *A. tumefaciens*, and external supplication of iron by 0.05 mM Fe_2_SO_4_·7H_2_O completely restored the biofilm-forming potential of *A. tumefaciens* (Figure 4B,C) growing with 100 or 200 µg/mL tannic acid. This suggests that tannic acid strongly chelates the iron and makes it unavailable for *A. tumefaciens* growth and significantly reduces its biofilm formation.

Tannic acid when present in the medium also inhibits the plant genetic transformation by *A. tumefaciens*. For example, varying levels of tannins released by roots of sorghum genotypes [58] have been suggested to be responsible for low *A. tumefaciens* inoculation efficiencies [59], which concurs with our SEM observations that tannic acid inhibited biofilm formation on the roots of *R. sativus* (Figure 6G–L) and inhibition of tumor formation by *A. tumefaciens* on potato slices (Figure 7). This reduction can be corroborated by a recent study by Torres et al. [60], where tannic acid was applied with aminoethylcysteine (chemical treatment) or *Pseudomonas protegens* CHA0 (biological treatment) to tomato roots infected with *A. tumefaciens* C58 derivative C107-KmR RifR. These chemical or biological combinations significantly reduced the LogCFU g^−1^ of *A. tumefaciens* C58 in root colonization and relative abundance [60].

In the present study, qRT-PCR revealed slight downregulation of the *dnaK* gene, which oversees bacterial stress response to tannic acid [61]. The VirA/VirG two-component system of *A. tumefaciens* is a prerequisite for wounded plant infection and tumor genesis, and cheV protein (encoded by the *chvE* gene) causes a sugar-induced increase in the expression of vir genes [13]. Furthermore, *chvE* mutation can create an imbalance between sugar utilization and consumption, which suggests the gene plays a major role in the control of *A. tumefaciens* virulence [62]. He et al. [62] reported that tannic acid downregulated the expression of *chvE* (responsible for sugar-induced increase in virulence), which suggested tannic acid reduced virulence. In addition, tannic acid targeting may be dependent on strain type; for example, tannic acid significantly repressed biofilm formations by methicillin-sensitive or resistant *S. aureus* by targeting the *ica* operon and several other regulatory genes [23], and we found tannic acid significantly downregulated the biofilm regulating the periplasmic regulator *exoR* gene by >15-fold (Figure 9). This gene appears to play a pivotal role in biofilm formation since *exoR* mutants failed to produce biofilms on polystyrene [39,63,64]. In addition, these mutants lost flagellar motility [63], which explained the reduced swimming motility of *A. tumefaciens* in the absence of any downregulation of genes directly governing motility. Moreover, ExoR may have multiple interacting partner proteins in periplasm and thus control other regulatory pathways [63].

## 5. Conclusions

This study demonstrates the polyphenol tannic acid efficiently prevents planktonic growth and biofilm formation of *A. tumefaciens* on abiotic (nitrocellulose and polystyrene) and plant root surfaces and suppresses virulence traits such as EPS secretion, protease activity, cell surface hydrophobicity, and swimming motility. Tannic acid remarkably inhibited biofilm formation on the surfaces of *R. sativus* roots without affecting *R. sativus* seed germination. One of the possible mechanisms of antibacterial and subsequent biofilm inhibition could be the chelation of iron by tannic acid due to the presence of galloyl groups. The other mechanism could be decreased expressions of *dnaK* and *chvE*, but significant downregulation of *exoR*, which codes for periplasmic succinoglycan-regulatory protein (a prerequisite of *A. tumefaciens* motility). This study improves the understanding of the interaction between tannic acid and *A. tumefaciens*. The results from this in vitro study are promising and pave the way for further research to assess the antipathogenic applications of tannic acid in agricultural settings with other plant species. Though pure tannic acid may inhibit the growth of other nontarget microbes; however, the possibility is low since some plants also release exudates having different tannins and still recruit beneficial microbes to the rhizosphere. Based on the results obtained, we propose that tannic acid be assessed for use in anti-*Agrobacterium* formulations under field conditions.

## Supplementary Materials

The following supporting information can be downloaded at: https://www.mdpi.com/article/10.3390/biomedicines10071619/s1, Table S1: Biofilm and virulence related genes of *A. tumefaciens* with their primer sequences used for qRT-PCR.
