# Supplementary material for: Inhibition of Agrobacterium tumefaciens Growth and Biofilm Formation by Tannic Acid
Source: Biomedicines. 2022 Jul 6;10(7):1619. doi: 10.3390/biomedicines10071619 (PMC9312696; doi:10.3390/biomedicines10071619)
Supplement: Supplementary file 1 [file biomedicines-10-01619-s001.zip › biomedicines-1782550-supplementary.pdf]

Supplementary Information

Supplementary Table S1: Biofilm and virulence related genes of *A. tumefaciens* with their primer sequences used for qRT-PCR.

Gene	Function	Primer sequences
16S rRNA	16S ribosomal RNA[1]	Forward 5′- TGA CGA GTG GCG GAC GGG TG -3′<br>Reverse 5′- ATG CAG TTC CCA GGT TGA GC -3′
celA	Cellulose synthase[2]	Forward 5′- TGC CAC AAA CAT CGA AAA AG -3′<br>Reverse 5′- ATT GTT CGG AAT TGG CAA AG -3′
cheA	Chemotaxis protein[2]	Forward 5′- GTG CCT TTG GTC TCG ATG AT -3′<br>Reverse 5′- GCT TCC AGT TCC TTG ACG AG -3′
exoR	Exopolysaccharide production negative regulator[2]	Forward 5′- CTT CTT TCC CTT GCC GAT TA -3′<br>Reverse 5′- CCT GGT TGA GCC ACT TCT TC -3′
phoB	Two component response regulator[2]	Forward 5′- AGG TTC TGT CTT CGG TGC TG -3′<br>Reverse 5′- TAG ATA TCG TGG CCC CAG AC -3′
chvE	Virulence factor kinase[3]	Forward 5′- GTT CTG CAG GCA ACT TCG AT -3′<br>Reverse 5′- ACC TTG TCC ATT CCC ATC TG -3′
chvG	Two component sensor kinase[3]	Forward 5′- GGA AAA ACT GCT CGA ATT GC -3′<br>Reverse 5′- CTG TCC GCG TGA ATA AAG GT -3′
flgE	Flagellar hook protein[4]	Forward 5′- TGG GAA GTA GCG GTT TAT CG -3′<br>Reverse 5′- GGT CGA TAT TGA TCG CTT GC -3′
fliR	Flagellar biosynthetic protein[4]	Forward 5′- GTC GTT GGC CTC ATC TTC TC -3′<br>Reverse 5′- TCC ATC ATG AAC AGC ACC AG -3′
motA	Flagellar motor protein[4]	Forward 5′- GAA GCG CAC ATC GAT AAT CC -3′<br>Reverse 5′- CGA TGA TGA TGA GAC GGA CA -3′
clpB	Protease[5]	Forward 5′- GCA AGC ATG TGG AGA AGG AT -3′<br>Reverse 5′- CAG AAA CCG GTC GGT AAT GT -3′
dnaK	Hsp70 chaperone protein[5]	Forward 5′- GTC GAC CAA CGG TGA TAC C -3′<br>Reverse 5′- GAT GAA CGG CAG GTT GAT TT -3′

[1]Housekeeping gene, [2]biofilm-related genes, [3]virulence genes, [4]motility genes, [5]stress response genes
